# Validation of a Novel Potentiometric Method Based on a Polymeric PVC Membrane Sensor Integrated with Tailored Receptors for the Antileukemia Drug Cytarabine

**DOI:** 10.3390/polym12061343

**Published:** 2020-06-14

**Authors:** Ayman H. Kamel, Abd El-Galil E. Amr, Nashwa H. Ashmawy, Hoda R. Galal, Abdulrahman A. Almehizia, Teraze A. Youssef, Mohamed A. Al-Omar, Ahmed Y. A. Sayed

**Affiliations:** 1 Chemistry Department, Faculty of Science, Ain Shams University, P.O. Cairo 11566, Egypt; ahkamel76@sci.asu.edu.eg (A.H.K.); nashwastar20@yahoo.com (N.H.A.); trease_albert@yahoo.com (T.A.Y.); 2 Pharmaceutical Chemistry Department, Drug Exploration & Development Chair (DEDC), College of Pharmacy, King Saud University, Riyadh 11451, Saudi Arabia; mehizia@ksu.edu.sa (A.A.A.); malomar1@ksu.edu.sa (M.A.A.-O.); ahmedyahia009@gmail.com (A.Y.A.S.); 3 Applied Organic Chemistry Department, National Research Center, Dokki, Giza 12622, Egypt; 4 Inorganic Chemistry Department, National Research Center, Dokki, Giza 12622, Egypt; hrgalal@hotmail.com

**Keywords:** cytarabine, molecularly imprinted polymers (MIPs), potentiometric sensors, antileukemia drug, method validation, pharmaceutical formulations

## Abstract

A simple, rapid and easy method is proposed for the detection of a cytostatic therapeutic drug, cytarabine, in real samples. The method is based on potentiometric transduction using prepared and characterized new ion-selective electrodes for cytarabine. The electrodes were integrated with novel man-tailored imprinted polymers and used as a sensory element for recognition. The electrodes revealed a remarkable potentiometric response for cytarabine over the linearity range 1.0 × 10^−6^–1.0 × 10^−3^ M at pH 2.8–4 with a detection limit of 5.5 × 10^−7^ M. The potentiometric response was near-Nernstian, with average slopes of 52.3 ± 1.2 mV/decade. The effect of lipophilic salts and plasticizer types on the potentiometric response was also examined. The electrodes exhibited an enhanced selectivity towards cytarabine over various foreign common ions. Validation and verification of the presented assay method are demonstrated by evaluating the method ruggedness and calculating the detection limit, range of linearity, accuracy (trueness), precision, repeatability (within-day) and reproducibility (between-days). The proposed ion-selective electrodes revealed good performance characteristics and possible application of these electrodes for cytarabine monitoring in different matrices. The electrodes are successfully applied to cytarabine determination in spiked biological fluid samples and in pharmaceutical formulations.

## 1. Introduction

Cytarabine, or cytosine arabinoside, is a pyrimidine nucleoside antimetabolite that is cytotoxic to a number of cell types. It is widely used for the treatment of acute leucosis, lymphoglanulomatosis, malignant lymphomas, etc. [1,2]. Cytarabine is considered as the main drug used in the treatment of acute leukemia, especially acute non-lymphocytic leukemia, when it is most often taken in combination with thioguanine and anthracycline. It is also given in the treatment of chronic myeloid leukemia and lymphoma, and it has been tried in the treatment of myeloma [3]. The quality of cytarabine preparations can be checked using different methods, including titrimetry [4], UV spectrometry [5,6,7], chemiluminescence [8], polarography [9,10,11,12,13,14], voltammetry [15], high performance liquid chromatography (HPLC) [16,17,18,19,20,21,22,23,24,25,26], gas chromatography (GC)/mass spectrometry [27,28,29], micellar electrokinetic capillary chromatography [30,31] and radioimmune assaying [32,33,34,35,36,37,38,39]. However, most of these methods are considered to have a high cost of use and suffer from limited selectivity and require careful control, whether in reaction conditions or even derivative reactions. These methods also require time-consuming treatment steps, which affect their usefulness for routine analysis. From all of the above, it is recommended to apply potentiometric sensors in the field of pharmaceutical analysis and biomedicine [40,41,42,43,44,45]. These sensors, based on potentiometric transduction, have many advantages, such as providing easy, simple, fast and selective technology to determine the number of different drugs in complex matrices [46,47,48,49,50]. However, with regard to the available literature, no one uses this technique for cytarabine quantification.

Integration of molecularly imprinted polymers (MIPs) in sensing technology enhances the performance of their use. These synthetic receptors have become a very important part in preparing synthetic and strong identification materials. They bring several advantages when coupled in potentiometric electrodes, especially in pharmaceutical analysis. They also offer enhanced selectivity for analytes (drugs) in different pharmaceutical or biological matrices when integrated with potentiometric devices [42,43,44,45,46,47,48,49].

In this work, the preparation, characterization and application of polymeric membrane sensors are presented on the basis of the use of cytarabine biomimetic receptors. The receptors were prepared by imprinting the template cytarabine with methacrylic acid (MAA) and ethylene glycol dimethacrylate (EGDMA) as a functional monomer and ethylene as a crosslinker, respectively. The MIPs were dispersed in a plasticized poly(vinyl chloride) (PVC) matrix membrane. The performance and selectivity characteristics of the proposed ion-selective electrodes (ISEs) were evaluated. Validation of the proposed method was evaluated in terms of linearity, detection limit, trueness, precision, accuracy, within-day repeatability and method ruggedness. The ISEs were successfully applied for cytarabine determination in spiked human serum samples and in pharmaceutical formulations.

## 2. Materials and Methods

### 2.1. Apparatus and Materials

All potentiometric measurements were measured and recorded at 25 ± 1 °C with an Orion 720/SA pH/mV meter (Cambridge, MA, USA). The measured potential was performed by immersing the proposed cytarabine electrode with an Ag/AgCl double-junction reference electrode (Orion, model 90-02) filled with a CH_3_COOLi reference electrode in stirred solutions. All experiments were performed with at least three electrodes. A glass electrode (Ross, Orion 81-02) was used for all subsequent pH measurements. All liquid chromatographic measurements were carried out using high-performance liquid chromatography (HPLC) coupled with a UV/VIS detector (Series 200 Pump, PerkinElmer, Waltham, MA, USA). For surface morphology measurements, scanning electron microscopy (SEM) (JOEL, Osaka, Japan) was used.

Cytosine β-D-arabinofuranoside hydrochloride (cytarabine), methacrylic acid (MAA) (functional monomer), potassium tetrakis (3,5-bis (trifluoromethyl) phenyl) borate (KTFPB) and acetonitrile were purchased from Sigma-Aldrich Inc. (St. Louis, MO, USA). Poly(vinyl chloride) (PVC) (polymeric matrix), ethylene glycol dimethacrylate (EGDMA) (crosslinker), benzoyl peroxide (BPO) (initiator), dioctyl phthalate (DOP), o-nitrophenyl octyl phthalate (o-NPOE), dibutyl sebacate (DBS) and tetrahydrofuran (THF) were obtained from Fluka AG (Buchs, Switzerland).

A 10^−3^-M cytarabine hydrochloride stock solution was prepared after dissolving 0.0243 g pure cytarabine hydrochloride in 100 mL distilled water. The pH of the solution was adjusted to pH 3.5 using a 30-mM phosphate buffer. Working cytarabine solutions (10^−3^–10^−7^ M) were prepared after appropriate dilution of the stock solution, and the pH of each working standard solution was adjusted to pH 3.5 using a 30-mM phosphate solution. The activity coefficients of all cytarabine solutions were calculated using the well-known approximation presented by Debye–Huckel.

### 2.2. Synthesis of the Main-Tailored Receptors

The artificial imprinted microbeads (MIPs) were tailored and designed for cytarabine using the thermal precipitation polymerization method. Prior polymerization, template cytarabine (0.5 mmol), MAA (1.5 mmol), EGDMA (1.5 mmol), acetonitrile (10 mL) and BPO (50.0 mg) were mixed together in a 25-mL glass-capped bottle and left for 1 h. For solution homogenization, the mixture was sonicated for 15 min. Dry N_2_ flow was passed in the solution for 15 min to get rid of the dissolved O_2_ in the solution. The reaction mixture was closed and maintained in an oil bath at 70 °C for 18 h for complete polymerization. The resulting MIP beads were washed with methanolic solution to remove all un-reacted species. For complete removal of the template from the MIP beads, Soxhlet extraction was carried out using acetic acid/methanol (2:8, v/v). Spectrophotometric measurements were carried out until the absorbance of cytarabine at 277.5 nm was no longer detected in the eluate [51]. The obtained polymers were left for complete dryness at ambient temperature. The non-imprinted polymers (NIPs) were also synthesized in absence of the template under the same conditions used for preparing the MIPs.

### 2.3. Binding Experiments

To examine the binding affinity of the prepared imprinted receptors towards cytarabine, binding experiments were carried out using Scatchard analysis. An amount of 20.0 mg of either MIP or NIP beads was mixed with 5.0 mL of cytarabine solution at different concentrations (0.02–1 mM) in a 10 mL conical flask. Shaking the polymeric beads in contact with the solution was carried out overnight at room temperature. The solution was centrifuged for 10 min to separate the polymeric beads from the supernatant. The free cytarabine concentration was detected spectrometrically at 277.5 nm. The data obtained were utilized for binding isotherms analysis.

### 2.4. Sensor Fabrication

An amount of 8.8 mg of either the MIP or NIP beads, 66.5 mg of PVC, 127 mg of the plasticizer (o-NPOE) and 2.2 mg of potassium tetrakis (3,5-bis (trifluoromethyl) phenyl) borate (KTFPB) were thoroughly mixed in a 3-cm diameter petri-dish and dissolved in 3 mL THF. The solvent was left to evaporate slowly at ambient temperature. The formed plastic membrane was sectioned with a cork borer (10 mm diameter) and glued to a PVC tube (~3 cm length, 8 mm i.d.) using THF. The body of the electrode consisted of a plastic tube attached to the PVC tube. This plastic tube was filled with an internal filling solution consisting of 10^−3^ M cytarabine hydrochloride solution.

Calibration of the proposed ISEs was carried out after transferring 1.0 mL aliquots of 10^−3^ to 10^−6^ M aqueous cytarabine hydrochloride solution to a 25-mL beaker, containing 9.0 mL 30 mM acetate solution (pH 3.5), followed by inserting both the cytarabine-PVC membrane sensor and the reference electrode. The obtained potential readings were plotted versus log (cytarabine) concentrations. The constructed calibration plots were used for all subsequent quantifications of unknown cytarabine concentrations.

### 2.5. Cytarabine Assessment in Pharmaceutical Dosages

Cytarabine was determined using the proposed ISEs in different commercially available drugs: aracytin (100 mg/5 mL, Pharmacia, Egypt), tabine (1000 mg/10 mL, Hikma Pharmaceuticals, Cairo, Egypt) and cytarabine (100 mg/mL, Ramco, Cairo, Egypt). One milliliter from each sample was placed into a 50-mL measuring flask and then completed to the mark with a 30-mM phosphate buffer solution at pH 3.0. The potential readings were recorded for each solution and then compared with the constructed calibration curve for the standard solutions of the pure drug under similar conditions.

### 2.6. Determination of Cytarabine in Spiked Human Serum

The applicability of the proposed sensors to quantify cytarabine in a complicated matrix was carried out. A human serum was chosen for this matrix and cytarabine was determined in spiked serum samples. An aliquot of human serum (3.0 mL) was placed in a 25-mL glass tube. A total of 9 mL of an ice-cold acetonitrile solution was added to the sample, and then the mixture was left for 5 min before centrifugation at 1000 rpm. The supernatant liquid, without removing any particles, was transferred to a 25-mL beaker and then left on a water bath at 50 °C until the volume was reduced to less than 3 mL. A total of 9 mL of 30 mM acetate buffer solution of pH 3.5 was added to the pretreated human serum sample. The mixture was thoroughly mixed and used for drug measurements. The sensor was immersed in conjunction with the reference electrode in the test solution. The potential readings were recorded after the equilibrium response was reached (10–20 s). The cytarabine concentration was calculated using a calibration graph.

## 3. Results and Discussion

### 3.1. MIP Characteristics

**Figure 1:**
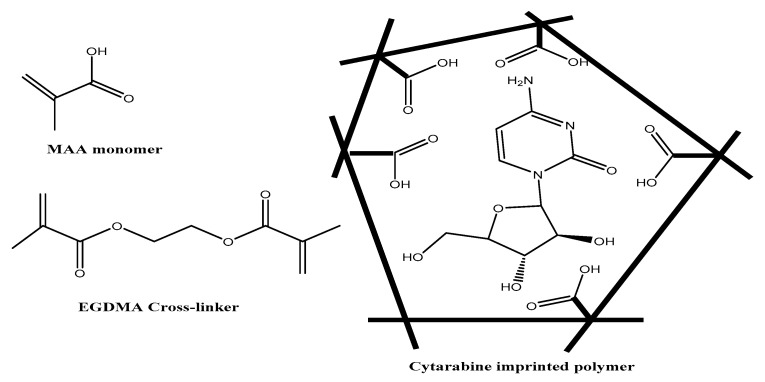
Schematic representation of the cytarabine imprinting process.

MIP-based electrochemical sensors come in various configurations that offer control of electrode properties, such as hydrophobic/hydrophilic character, permeability and film thickness. Control of all of these properties is essential for excellent sensor performance. One of the main technical challenges in developing inexpensive MIP-based sensors is to achieve an appropriate interface between the recognition element (MIPs) and the transducer. In most cases, the MIPs should be in close contact with the transducer surface. A schematic presentation of the MIP progressive manufacturing process is shown in Figure 1.

**Figure 2:**
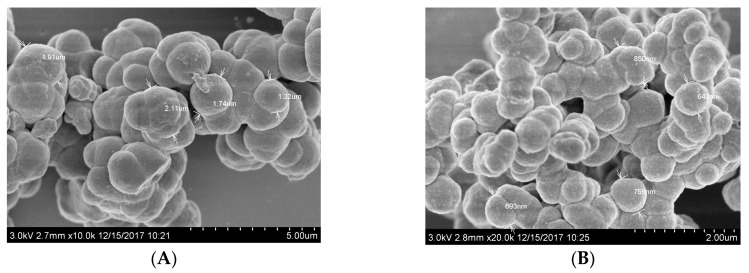
SEM images for both (**A**) cytarabine molecularly imprinted polymer (MIP) and (**B**) non-imprinted polymer (NIP) beads.

Scanning electron microscopy (SEM, JOEL, Osaka, Japan) was utilized to check the surface morphology of the prepared polymers, either MIPs or NIPs. As shown in Figure 2, the cytarabine imprinted beads in addition to the NIPs were found to be uniform and spherical in shape, with an average diagonal distribution of 1.32–2.11 µm and 0.69–0.85 µm, respectively. This difference in the particle size can be ascribed to the existence of cytarabine molecules as a template. Because of this spherical size and shape, well-obtained beads can propagate into the ISE membrane and reveal a high-binding ability towards the cytarabine molecule. The good distribution of the polymeric beads in the polymeric membrane offers more available recognition and binding sites in the sensing membrane of the sensor and results in a better response performance.

### 3.2. Binding Features of the Prepared MIPs

The site distribution and binding mode between the prepared MIPs and cytarabine were evaluated using binding experiments. Fixed amounts of the imprinted polymers were incubated with different concentrations of cytarabine until equilibrium was achieved. The binding capacity of the MIPs (*Q*) was calculated according to Equation (1):

*Q* = *µmol* (*bound cytarabine*)/*g*(*MIP*) (1)

**Figure 3:**
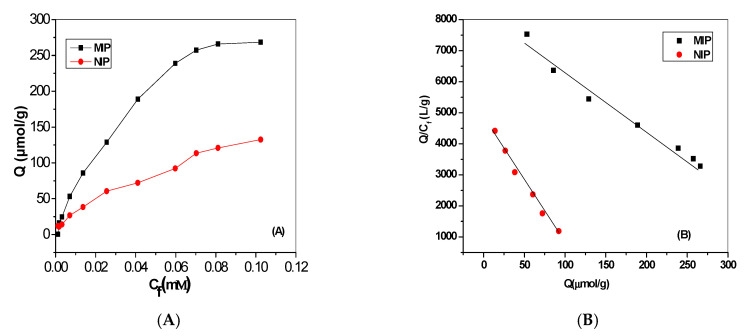
Binding characteristics of the prepared beads: (**A**) binding isotherms and (**B**) Scatchard plot.

The binding capacity at each cytarabine concentration was plotted against the initial cytarabine concentration. As shown in Figure 3A, the binding capacity increased with an increase in the initial concentration of cytarabine until reaching saturation at higher analyte concentrations. Further binding parameters, such as maximum binding capacity (*Q_max_*) and the equilibrium dissociation constant at the binding sites (*Kd*), were evaluated using the Scatchard equation:

*Q*/*C_free_* = (*Q_max_* − *Q*)/*K_d_* (2)

where *C_free_* is the free cytarabine concentration at equilibrium (mmol/L). As shown in Figure 3B, the Scatchard plot was linear over the entire cytarabine concentration range. This indicates the presence of homogeneous bonding sites. The equilibrium dissociation constants, *K_d_* for both MIP and NIP beads were 0.054 and 0.024 µmol/L, respectively. The apparent maximum amounts, *Q_max_,* for both MIP and NIP beads were found to be 444.9 and 117.7 µmol/g, respectively. From the abovementioned results, the MIPs combined a small dissociation constant with a high-maximum apparent binding capacity, as compared to the NIPs. This confirms the strong binding affinity of the MIPs and their successful use as an ionophore towards cytarabine.

### 3.3. Response Characteristics of the Proposed Sensors

**Figure 4:**
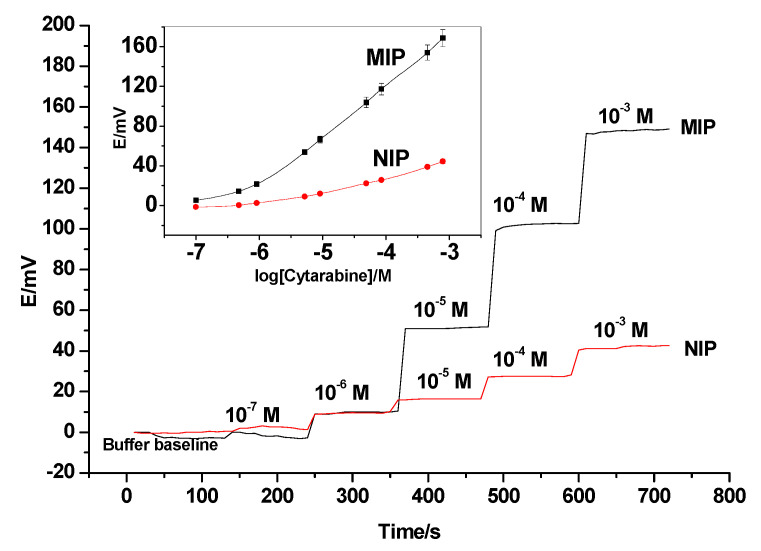
Potentiometric behavior of the cytarabine-membrane-based sensor (time trace and potentiometric response).

Liquid-contact cytarabine sensors based on potentiometric transduction were fabricated. The sensing membranes were based on MIP or NIP beads, dispersed in a plasticized PVC polymeric matrix. The designed sensors were characterized for the potentiometric assay of cytarabine according to International Union of Pure and Applied Chemistry (IUPAC) guidelines [52]. Sensors based on MIP beads dispersed in different plasticizers (i.e., o-NPOE, DOP and DBS) from triplicate studies (*n* = 3) revealed a near-Nernstian slope of 52.3 ± 1.2 (*R*^2^ = 0.999), 48.3 ± 1.1 (*R*^2^ = 0.998) and 45.6 ± 0.9 (*R*^2^ = 0.998) mV/decade, with detection limits of 5.5 × 10^−7^, 8.2 × 10^−7^ and 1.2 × 10^−6^ M, respectively. However, sensors based on non-imprinted polymer (NIP) particles showed no response towards cytarabine due to the lack of binding receptors in the membrane. The sensors revealed a sub-Nernstian slope of 18.4 ± 1.6 (*R*^2^ = 0.995) mV/decade, with a detection limit of 3.2 × 10^−6^ M. The performance response characteristics are tabulated in Table 1. The obtained potential response of these sensors is shown in Figure 4.

### 3.4. Method Validation

The quality and consistency of the data obtained were verified by the inserted electrodes according to international standard guidelines for validation of the analytical methods, including ISO/IEC 17025/2017, Association of Official Analytical Chemists (AOAC), United States Pharmacopeia (USP), United States Environmental Protection Agency (US EPA),World Health Organization (WHO) and U.S. Food and Drug Administration (US FDA) [53,54]. Method validation elements were evaluated using six batches (triplicate each) of standard cytarabine solution. These elements include method linearity, limit of detection, trueness (accuracy and bias), standard deviation (precision), robustness and interference study.

#### 3.4.1. Method Linearity and Detection Limit

Method linearity was verified by measuring cytarabine standard solution in the range of about 0.243 to 243.2 μg/mL. From the constructed calibration plot, the linear range was 1.0 × 10^−3^–1.0 × 10^−6^ M (243.22–0.243 μg/mL) for the MIP-based membrane sensors. Equation (3) shows the least square analysis of the data:

*E* (*mV*) = (52.3 ± 1.2) *log* [*Cytarabine*] + (330.5 ± 2.1) (3)

The statistical analysis for the linearity data is tabulated in Table 1. The limit of detection was measured from the intersection of extrapolated linear stable portions of the calibration graph, according to IUPAC guidelines [52]. It was calculated and found to be 5.5 × 10^−7^ M (0.13 μg/mL). Examining any fixed or relative bias caused by the proposed electrodes, a simple linear regression of the observed concentrations against the expected values (five points) was performed. No remarkable change in the slopes of the regression lines was observed, and the correlation coefficients (*R*^2^) were nearly the same as those of the ideal value of unity (*R*^2^ = 0.999). The change in the intercepts was shown to be very small. No systematic difference was recorded between the estimated and expected concentrations within the tested range using the presented method.

#### 3.4.2. Accuracy and Precision

The method accuracy was estimated by spiking a known amount of cytarabine to urine samples as a blank matrix at concentration levels 10–20 μM. All sample solutions were analyzed as per the proposed method in triplicate. The percentages of recovery from the matrix were 99.1%, 98.8% and 98.6% for 10, 15 and 20 μM cytarabine concentrations, respectively.

The term precision demonstrates the repeatability and reproducibility of the method and is expressed as the coefficient of variation (CV). It was achieved by performing six replicate introductions of cytarabine standard solutions (10, 15 and 20 μg/mL) into the proposed system. The relative standard deviations were calculated and found to be 0.7%, 0.9% and 1.1% for each concentration, respectively.

#### 3.4.3. Method Robustness and Ruggedness

Method robustness was evaluated by testing the effect of changing the pH of the test solution and measuring the response time of the proposed electrodes. The effect of the solution pH on the potentiometric response of the presented electrodes was examined. The potential response offered by standard 1.0 × 10^−4^ M cytarabine was recorded over a pH range of 2–11. Adjustment of the test solution was carried out using either HCl and/or NaOH solutions. It was noticed that there was no change in the potential response of this concentration over the range of 2.8–4.0. Since the p*Ka* of cytarabine is 4.22 [55], one pH unit below the pKa resulted in 99.9% ionization (protonation) of the drug. At pH 4, the potential response declined with negative drift due to the formation of the free base of the drug. A 30-mM phosphate buffer at pH 3 was used as a solution background for all subsequent measurements.

The dynamic time response of the proposed electrodes was also tested. Stable and constant potential was attained within ~5–10 s for 10^−3^–10^−6^ M cytarabine test solutions to reach an approximated 95% equilibrium response (Figure 4). The method ruggedness was examined by performing the analysis using six different electrodes and two different instruments on different days. Repeatability (within-day) and reproducibility (between-days) showed a difference in potential within 2–3 mV. The obtained data showed that the effect of the studied parameters falls within the specified tolerance, and the changes are considered within the method robustness.

#### 3.4.4. Interfering Study

Potentiometric selectivity coefficients (*K^pot^_i,J_*) of cytarabine sensors were carried out through the modified separate solutions method (MSSM) proposed by Bakker [56]. The selectivity values reflect the preferential interaction of the tailored receptors with cytarabine in a 50-mM phosphate buffer solution of pH 3. The selectivity over various related compounds and inorganic ions (e.g., K^+^, Na^+^, Ca^2+^, Mg^2+^, fluoxetine, metformine, caffeine, pheniramine, creatine, glutamine, creatinine, histidine and quinine) is presented in Table 2.

Pharmacological excipients, such as glucose, maltose, starch, talc and tween-80 used at a concentration level less than 1000 times above cytarabine, have no effect on the result accuracy obtained by the proposed electrodes.

### 3.5. Analytical Applications

Cytarabine membrane sensors were successfully introduced to monitor the amount of cytarabine in different pharmaceutical formulations.

It can also be used for routine analysis and quality control/quality assurance during drug manufacture. Potentiometric assaying of cytarabine in triplicate showed results with an average recovery of 99.3% and a mean standard deviation of ±0.5% (Table 3). The data were compared with results obtained by liquid chromatography with detection at 254 nm [57]. An *F*-test demonstrated that there was no difference between the means and the measured variances of the two outcome groups of the results. The distribution of measurements and the extent of determination under investigation indicate that the results are subject to statistical control.

The sensors were successfully tested monitoring cytarabine in human serum by spiking aliquots of different samples with known amounts of standard cytarabine solution. The recovery results showed an average of 99.1% with a relative standard deviation of ±0.8%. The results obtained for the determination of cytarabine are presented in Table 4.

## 4. Conclusions

Novel liquid-contact cytarabine potentiometric sensors were designed for the quantification of the antileukemia cytarabine drug in different pharmaceutical formulations. The membrane sensors were based on the use of tailored imprinted polymers as recognition receptors. The prepared MIPs were based on the use of MAA as a functional monomer and EGDMA as a crosslinker. The sensors provided the advantages of rapid response, reasonable selectivity, elimination of premedication or separation steps, low cost, long-term stability, good selectivity and automatic feasibility. Advantages offered using the proposed cytarabine sensor are the low detection limit (5.5 × 10^−7^ M), long-life span (8 weeks) and extended linearity range (10^−3^–10^−6^ M). The validity of the proposed method was verified by measuring the lowest limit of detection, range, accuracy, precision, repeatability and variability between days. The good performance characteristics obtained demonstrate the applicability to the determination of cytarabine in different drug formulations. The method can be successfully applied for the routine control of pharmaceutical drug solutions without any pre-concentration procedures.

## Figures and Tables

**Table 1:** Response characteristics of the proposed cytarabine sensors.

Parameter	MIP Membrane Based Sensor			NIP Membrane Based Sensor
	o-NPOE	DOP	DBS	
**Slope (mV/decade)**	52.3 ± 1.2	48.3 ± 1.1	45.6 ± 0.9	18.4 ± 1.6
**Correlation coefficient (R^2^)**	0.999	0.999	0.998	0.995
**Detection limit (M)**	5.5 × 10^−7^	8.2 × 10^−7^	1.2 × 10^−6^	3.2 × 10^−6^
**Linear range (M)**	1.0 × 10^−6^–1.0 × 10^−3^	2.8 × 10^−6^–1.0 × 10^−3^	5.0 × 10^−6^–1.0 × 10^−3^	1.0 × 10^−5^–1.0 × 10^−3^
**Working pH range (pH)**	2.8–4.0	2.8–4.0	2.8–4.0	-
**Response time (s)**	<10	<10	<10	-
**Accuracy (mV%)**	99.2	98.6	98.1	-
**Precision (mV%)**	0.7	1.1	2.2	-

**Table 2:** Potentiometric selectivity coefficients (*log*$k_{i,j}^{pot}$) of the cytarabine membrane sensor plasticized with o-nitrophenyl octyl phthalate (o-NPOE) in a 50-mM phosphate solution of pH 3.0.

Interfering Ion	−log *K ^pot^_I, J_*
**Na^+^**	5.1
**K^+^**	4.7
**Ca^2+^**	5.7
**Mg^2^**	5.8
**Fluoxetine**	5.0
**Metformine**	4.2
**Caffeine**	4.9
**Pheniramine**	4.3
**Creatine**	5.1
**Glutamine**	4.1
**Creatinine**	4.4
**Histidine**	3.8
**Quinine**	3.9

**Table 3:** Cytarabine assessment in different drug formulations using the cytarabine-membrane-based sensor.

Pharmaceutical Product and Source	Nominal Content Taken, mg /mL	Found, mg Tablet^−1^				*t*-Student Test ^b^	*F*-Test ^b^
		Proposed Method	Mean ^a^ (%) ± SD	Reference Method [57]	Mean ^a^ (%) ± SD		
**Aracytin (Pharmacia, Egypt)**	20	20.3	101.5 ± 0.4	20.1	100.8 ± 0.6	1.2	4.2
**Tabine (Hikma pharmaceutical, Egypt)**	100	99.5	99.5 ± 0.6	99.9	99.9 ± 0.1	3.2	7.6
**Cytarabin (Ramco, Egypt)**	100	98.8	98.8 ± 0.5	99.8	99.8 ± 0.1	2.3	3.4

^a^ Mean of triplicate measurements ^b^ Student’s *t*-test and *F*-test at 95% confidence level values are 4.30 and 19.00, respectively.

**Table 4:** Cytarabine assessment in human serum using the proposed sensors.

Sample	Spiked, μg/mL	*Found, μg/mL	Recovery, %	RSD, %
1	10	9.7	97.0	1.1
2	15	14.3	95.3	0.8
3	20	20.6	103.0	0.9

* average of 5 measurements.
